# Ornithophily in the trumpet creeper (*Campsis radicans*)

**DOI:** 10.1002/ece3.70279

**Published:** 2024-09-10

**Authors:** Gary R. Graves

**Affiliations:** ^1^ Department of Vertebrate Zoology, MRC‐116, National Museum of Natural History Smithsonian Institution Washington DC USA; ^2^ Center for Macroecology, Evolution, and Climate, Globe Institute University of Copenhagen Copenhagen Ø Denmark

**Keywords:** *Archilochus colubris*, *Campsis radicans*, ornithophily, ruby‐throated hummingbird, trumpet creeper

## Abstract

The diversification of hummingbirds (Trochilidae) has shaped the pollination strategies and floral trait evolution in at least 68 families of flowering plants in the Western Hemisphere. The trumpet creeper (Bignoniaceae) is the quintessential example of ornithophily in eastern North America. The mutualistic relationship between this orange‐flowered liana and the ruby‐throated hummingbird (*Archilochus colubris*) was illustrated as early as 1731. However, neither historical nor modern accounts accurately describe the feeding behavior of ruby‐throats at trumpet creeper flowers or the floral adaptations for ornithophily. This paper explores their surprisingly immersive mode of foraging at trumpet creeper flowers and quantitatively assesses floral traits in two populations in the Ozark Mountains. The ruby‐throat's bill is approximately one‐third the length of the trumpet‐shaped flowers, which counters the tendency for the corolla length of ornithophilous plants to match the bill length of their principal hummingbird pollinator. To access the nectary, ruby‐throats grasp or cling to the ventral petal lobe of the corolla with their claws and thrust their head and upper body into the flower. This immersive “floral‐diving” had not been formally documented among the 356 species of hummingbirds until now. The didynamous anthers and stigma are strategically positioned inside the corolla to brush the crown feathers when the ruby‐throat inserts its head. A narrow stricture in the corolla, about a third of the way up, allows the bill and tongue of hummingbirds to pass while blocking bumblebees and carpenter bees from reaching the nectary. As a result, the abundant sucrose‐rich floral nectar seems to be reserved for hummingbird pollinators.

## Graphical abstract

The trumpet creeper (*Campsis radicans*) is the prime example of ornithophily in eastern North America. This paper describes the unique foraging behavior of its principal pollinator, the ruby‐throated hummingbird (*Archilochus colubris*), and presents a quantitative analysis of floral adaptations for ornithophily in the Ozark Mountains.

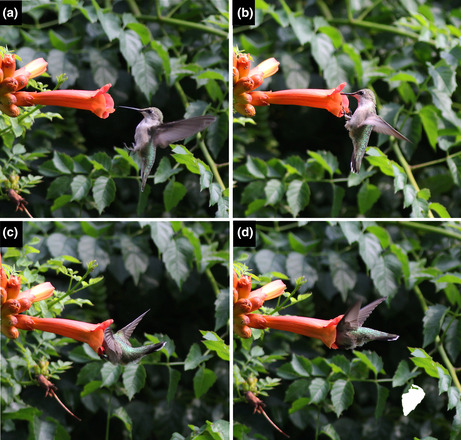

## 1 INTRODUCTION

Ornithophily refers to the evolutionary adaptation of flowering plants for bird pollination (Cronk & Ojeda, 2008; Faegri & Van Der Pijl, 1979). Hummingbirds (Trochilidae) are the most specialized nectarivorous birds in terms of morphology and behavior (Schuchmann, 1999; Stiles, 1978). This monophyletic clade, with a peak in species diversity in the equatorial Andes (Rahbek & Graves, 2000), has been diversifying in the Western Hemisphere for at least 22 million years (McGuire et al., 2014). The 356 extant species (Chesser et al., 2022; Remsen et al., 2023) occupy a collective geographic range spanning 115 degrees of latitude from Alaska to Tierra del Fuego. The trochiliform radiation has had a profound impact on the floral evolution and pollination strategies of flowering plants (Barreto et al., 2024; Grant & Grant, 1968; Temeles & Kress, 2003). An estimated 7000 plant species representing 68 plant families are reliant on hummingbirds for pollination, the dependence inferred from generic‐level characters such as brightly colored, scentless flowers with elongated corolla tubes, exposed stigmas and stamens, and the production of large volumes of sucrose‐rich nectar (Abrahamczyk et al., 2014). It is conceivable that the number of hummingbird‐pollinated species exceeds that of all other bird‐pollinated plants worldwide.

The flora of eastern North America includes 31 plant species (Austin, 1975; Bertin, 1982c), representing 21 genera and 18 families, that are pollinated by the ruby‐throated hummingbird, *Archilochus colubris* (L.), the sole breeding hummingbird in this region (Weidensaul et al., 2020). Trumpet creeper (*Campsis radicans* (L.) Seem. ex Bureau), a member of the cosmopolitan Bignoniaceae, is the quintessential example of ornithophily in the eastern United States. This flamboyantly orange‐flowered liana is widely distributed east of the Great Plains, particularly south of 40°N latitude (Kartesz, 2015), with a native geographic range greater than 1.5 million km^2^. Thriving in sunny locations on moist rich soils, especially in riparian corridors, trumpet creeper frequently climbs trees to a height of 15 m and sprawls over fencerows, embankments, masonry walls, bridge abutments, and abandoned buildings. The zygomorphic flowers (60–90 mm in length) are arranged in terminal inflorescences composed of 12–35 flowers organized in 3‐flowered dichasial cymes (Bertin, 1982a; Gentry, 1992). Mature plants may bear hundreds of blossoms during the summer flowering season.

The foundational studies on the pollination ecology of trumpet creeper began in the 1970s (Bertin, 1982a, 1982b, 1990a, 1990b; Bertin & Peters, 1992; Bertin & Sullivan, 1988; Elias & Gelband, 1975, 1976; Hardin et al., 1972). Working at field sites in Missouri and Illinois, Bertin (1982a) confirmed that the liana was adapted for hummingbird pollination. Although bumblebees and honeybees were capable of pollinating flowers, ruby‐throats deposit ten times as much pollen per visit. More recent studies in Tennessee, where hummingbirds were scarce (Van Nest et al., 2021), and in Poland, where trumpet creeper was introduced as a garden ornamental and hummingbirds are absent (Kolodziejska‐Degorska & Zych, 2006), confirmed the efficacy of bee pollination. The presence of contrasting nectar guides inside the corolla (Hardin et al., 1972), a characteristic of melittophilous flowers, suggests that trumpet creeper may employ a hybrid pollination strategy to attract both hummingbirds and bees.

Trumpet creeper has been admired by horticulturists since the Colonial era for its showy flowers (Cothran, 2003). It was among the first ornamental plants from North America introduced to Europe, with records of its cultivation in England dating to 1640. It has become naturalized far outside its native range in North America, Asia, South America, and Europe (Observations iNaturalist). Due to its aggressive growth and clonal spreading, it can quickly become unmanageable in gardens and ornamental plantings. Trumpet creeper has been classified as one of the more problematic native weeds in the southeastern United States (Elmore et al., 1989; Marble, 2018).

### 1.1 Historical prolog

**FIGURE 1:**
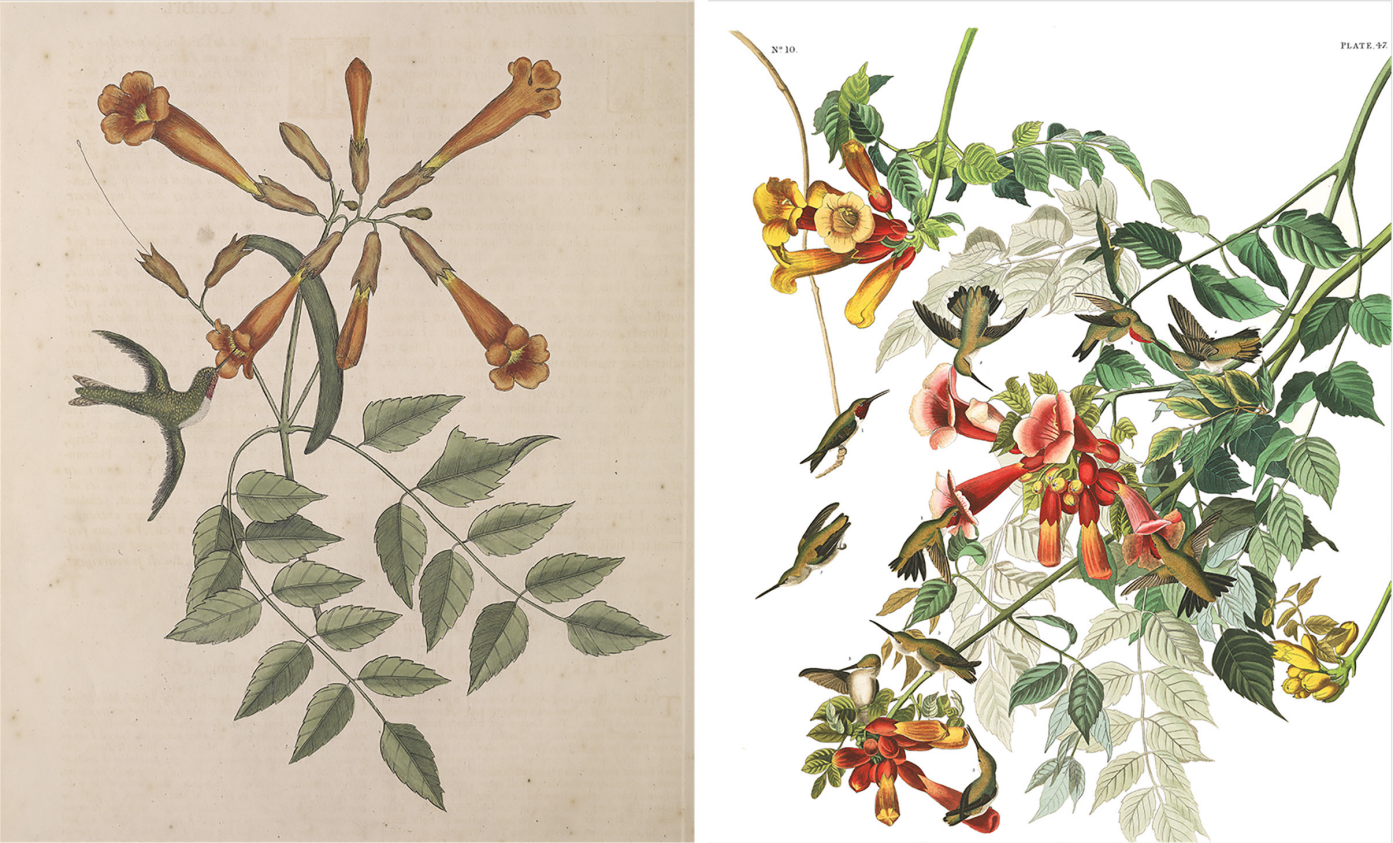
Illustrations of ruby‐throated hummingbird (*Archilochus colubris*) and trumpet creeper (*Campsis radicans*): Left by Catesby (1731) and right by Audubon (1835).

The ruby‐throat is the most intensively researched hummingbird species (Schuchmann, 1999; Weidensaul et al., 2020), with a historical literature dating to the 17th century (Clayton, 1693; Grew, 1693; Josselyn, 1672). The earliest known illustration of an ornithophilous plant with its primary pollinator (Figure 1) is a hand‐colored etching of a ruby‐throat feeding at a trumpet creeper flower (Catesby, 1731). Early natural history accounts mentioned several nectar plants (Bartram, 1792; Kalm, 1770), with trumpet creeper receiving the most attention (Audubon, 1831, 1835; Catesby, 1731; Nuttall, 1832; Wilson, 1810). The mutualistic relationship between trumpet creeper and ruby‐throats was well known several decades before the formal conceptualization of ornithophily as a pollination syndrome in flowering plants (Delpino, 1867).

Catesby (1731: 65) appears to be the only observer to note the considerable physical exertion required for these birds to obtain nectar legitimately from trumpet creeper flowers, “The Humming Birds delight to feed on these Flowers; and, by thrusting themselves too far into the Flower, are sometimes caught.” This observation was presumably made in the Carolinas or Virginia.

Alexander Wilson, who had access to Catesby's volume (Ord, 1828), composed a lengthy account on ruby‐throats in his classic *American Ornithology*, which included a description of it feeding at trumpet creeper flowers (Wilson, 1810: 28):

The Humming‐bird is extremely fond of tubular flowers, and I have often stopt, with pleasure, to observe his manoeuvres among the blossoms of the trumpet flower. When arrived before a thicket of these that are full blown, he poises, or suspends himself on wing, for the space of two or three seconds, so steadily, that his wings become invisible, or only like a mist; and you can plainly distinguish the pupil of his eye looking round with great quickness and circumspection; the glossy golden green of his back, and the fire of his throat, dazzling in the sun, form altogether a most interesting appearance. The position into which his body is usually thrown while in the act of thrusting his slender tubular tongue into the flower, to extract its sweets, is exhibited in the figure on the plate [X].

Wilson's account made no mention of ruby‐throats inserting themselves in flowers or becoming trapped. It is plausible that he either overlooked Catesby's observations or did not fully appreciate their significance.

Audubon (1831), who closely studied Wilson's work, did not describe the mode of ruby‐throat foraging at trumpet creeper flowers. However, his iconic painting of ruby‐throats (1835) showed a swarm feeding at a cluster of trumpet creeper inflorescences. Two birds are depicted with their bills inserted in flowers, although not deeply enough to obtain nectar. Both are hovering rather than perched, giving the impression that ruby‐throats are capable of legitimately obtaining nectar from trumpet creeper flowers without fully inserting their heads inside the corolla. This portrayal became the prototypical representation of ruby‐throat foraging behavior and likely shaped the perception that further study was unnecessary. As a result, neither historical nor modern accounts accurately describe their feeding behavior at trumpet creeper flowers.

This paper unravels the mystery of Catesby's “caught” hummingbirds and documents an immersive foraging mode in ruby‐throats that is, at the very least, rare among hummingbirds. An analysis of floral traits further clarifies the ornithophilous adaptations of trumpet creeper for hummingbird pollination.

## 2 METHODS

### 2.1 Trumpet creeper populations

I investigated floral traits at two locations 20 km apart in Baxter County, Arkansas, near the southern margin of the Salem Plateau of the Ozark Mountains. These sites lie in the western portion of the liana's native range, (Observations · iNaturalist), approximately 215 km southwest of Bertin's (1982a) nearest study area in Missouri.

- White River (36°11.7′ N, 92°16.9′ W; elevation 110 m). Flowers were sampled from July 29, 2023, to September 20, 2023, from two intertwined lianas growing on an arbor situated on sandy loam on the bank of the White River, the principal river in the Ozark highlands.
- Norfork Lake (hereafter Norfork) (36°22.5′ N, 92°13.9′ W; elevation 177 m). Flowers were sampled from August 4, 2023, to September 20, 2023, from numerous lianas growing along 75 m of roadside tangles on a well‐drained cobbled slope overlooking the lake, an impoundment of the North Fork of the White River.

### 2.2 Overview of trumpet creeper floral traits

**FIGURE 2:**
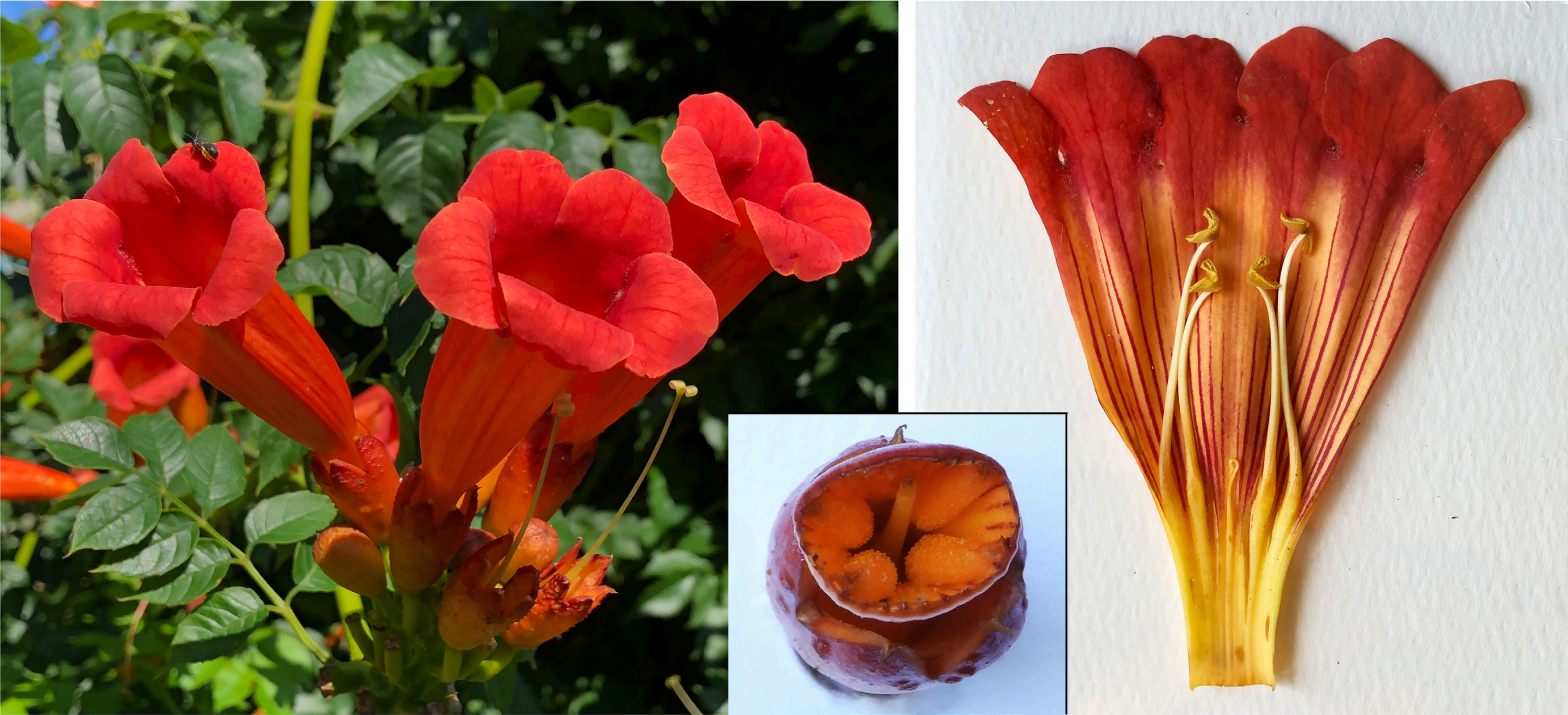
Trumpet creeper flowers. Left: Cluster of flowers in typical upright orientation. Right: Corolla split along the ventral midline. The ovary, nectary, and style have been removed with the calyx. Inset: View of the corollary stricture after the distal end of the flower can be removed. The severed stump of the style blocks the center of the narrow aperture (2–3 mm wide).

The campanulate corolla widens from a narrow elongated cylindrical base and terminates in five rounded petal lobes that unfold at anthesis. The ventral petal lobe (Figure 2) functions as a landing platform for hummingbirds and bees. The pentagonal corolla opening is vertically compressed, wider horizontally than it is tall. The base of the corolla, which encloses the basal nectary and superior ovary, is protected by a leathery cup‐shaped calyx. Most corollas curve subtly downward above the calyx. The circular nectary averages 4.0 mm in diameter (Elias & Gelband, 1976). The corollary stricture marks the point where the narrow basal tube flares abruptly. The four staminal filaments and a short curled staminode emerge from the interior wall at the corollary stricture and curve along the inner walls toward the opening. The anthers are arranged in a didynamous configuration, with a proximal and distal pair. The dorsifixed anthers are positioned along the dorsal wall inside the corolla opening. The style protrudes through and partially blocks the aperture of the stricture (Figure 2). The flattened, approximately diamond‐shaped stigma usually lies between the proximal and distal pairs of anthers along the dorsal wall of the floral tube.

Trumpet creeper flowers contain an anthocyanin pigment, cyanidin 3‐rutinoside (Harborne, 1967), resulting in rich orange and red hues on the internal and external surfaces of the corollas (Figure 2), typical of hummingbird‐pollinated flowers (Grant & Grant, 1968). Contrasting red to magenta nectar guides extend from the reflexed petal lobes at the mouth of the corolla inward past the anthers and stigma toward the corollary stricture (Figure 2).

Individual flowers can yield nectar volumes exceeding 115 μL/day (Bertin, 1982a; Edge, 2010; Van Nest et al., 2021), the highest average volume reported for ornithophilous plants in North America at temperate latitudes (see McDade & Weeks, 2004; Stiles, 1975; Stiles & Freeman, 1993). Flowers typically open between 06:00 and 14:00 h (Bertin, 1982a). Newly opened flowers have an average initial nectar volume of 40 μL. Nectar production continues at a rate of 5 μL/h throughout the day, slows abruptly at sunset, and ceases the following day (Bertin, 1982a). Trumpet creeper flowers, like those of most ornithophilous species, have little or no scent, at least to human olfaction. The sugar concentration of floral nectar, in sucrose equivalents, ranges from 24% to 35%, averaging approximately 30% (Bertin, 1982a; Edge, 2010; Van Nest et al., 2021). This concentration is slightly higher than the average 25.4% sugar observed in 202 species of hummingbird‐pollinated plants but significantly lower than the average 41.6% observed in 156 bee‐pollinated plants (Pyke & Waser, 1981). Floral nectar sugar of trumpet creeper is predominately sucrose (73%), with lesser percentages of fructose (18%) and glucose (9%) (Baker et al., 1998). In a sample of 137 hummingbird‐pollinated species, Baker et al. (1998) found a range of 18–97% sucrose, with an average of 58%. Extrafloral nectaries have been identified on the petiole, calyx, and the outer surface of developing corollas (Elias & Gelband, 1975, 1976). These are visited by ants. There is no evidence that hummingbirds exploit extrafloral nectar, which must be miniscule in calorie equivalents compared to the copious floral nectar.

### 2.3 Floral data

I stored newly opened flowers in plastic bags and transported them in a refrigerated cooler for later measurement and dissection. Insect‐damaged flowers were culled in the field, while undersized flowers were retained in the population samples. Most floral measurements were made with digital calipers to the nearest 0.1 mm. The width of the corolla opening (horizontal plane) was measured first. The corolla was then split distally along the ventral midline far enough to measure the position of the stigma in relation to the proximal and distal pairs of anthers. The calyx, nectary, ovary, and stigma were then removed as a single unit. Corolla length (minus calyx) was measured from the base, which approximates the position of the nectary, to the notches flanking the ventral petal lobe at the mouth of the floral tube. The corolla was then split from end to end along the ventral midline, pressed flat under a sheet of glass, and photographed alongside a scale bar (Figure 2). The distance from the corolla base to the corollary stricture, approximated by the base of the distal anther filaments, was measured from scaled photographs (to the nearest mm). I also measured the distance from the corolla base to the filament tips of the longest proximal and distal pairs of anthers. The stigma‐nectary distance was derived from the position of the stigma relative to the distal pairs of anthers.

### 2.4 Hummingbird foraging behavior

I observed ruby‐throats foraging at the White River site during three flowering seasons: July 9, 2020, to August 8, 2020, June 30, 2021, to August 11, 2021, and July 29, 2023, to September 20, 2023. Hummingbirds at this site were habituated to humans and commonly fed at trumpet creepers within 2 m of seated observers. None of the hummingbirds was marked or tagged. Video recordings of foraging were made with a Nikon Coolpix P1000™ camera. Still photography was also conducted with a Canon Eos 80D™ equipped with a 400 mm lens.

### 2.5 Statistical analysis

The pollination biology literature provides limited guidance on the expected statistical distribution of floral trait measurements in ornithophilous plants. To address this gap, I calculated summary statistics for six key floral measurements that bear directly on hummingbird pollination and nectar extraction in trumpet creeper (Appendix 1). The D'Agostino‐Pearson *K* ^*2*^ test (hereafter *K* ^*2*^ test), which combines the skewness ($\sqrt{b_{1}}$) and kurtosis (*b*_*2*_) statistics, was used to evaluate the goodness‐of‐fit of floral trait measurements to a normal distribution (D'Agostino et al., 1990). The *K* ^*2*^ test has robust power over a wide range of sample sizes and is preferred over the Shapiro–Wilk *W* test (Shapiro & Wilk, 1965) when data sets include repeated values. I report φ coefficients as a measure of the degree of similarity between observed distributions and a normal distribution. The values of *φ* range from −1 to 1, where zero indicates perfect conformity to a normal distribution.

**FIGURE 3:**
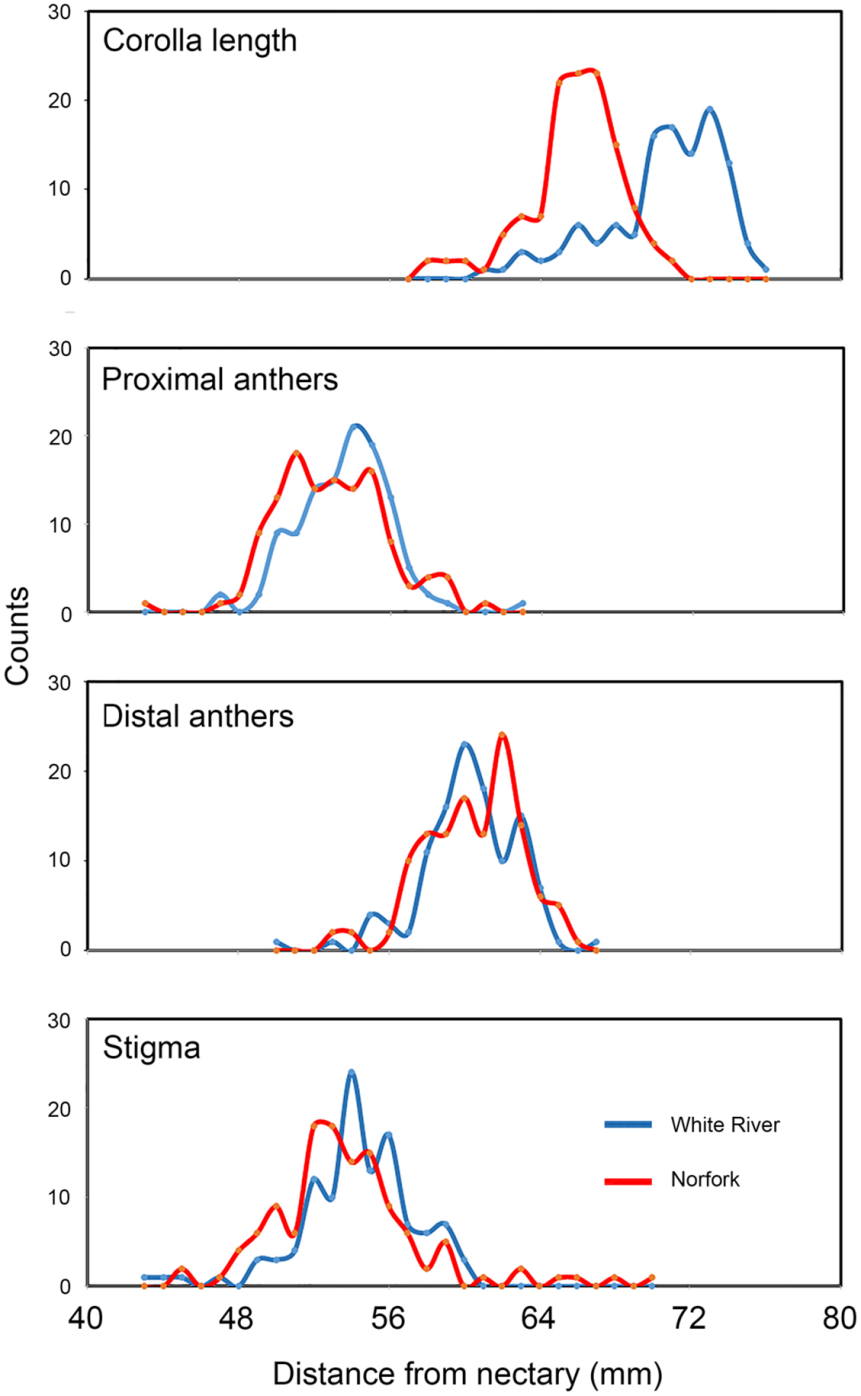
Trait distributions of trumpet creeper flowers at two sites in the Ozark Mountains. Corolla length and the distances from the floral nectary to the proximal anthers, distal anthers, and stigma. These data document the inserted positions of the reproductive structures (Appendix 1).

None of the six floral traits displayed significant skewness (Appendix 1). Kurtosis was observed in a single trait in the White River population. However, the omnibus *K* ^2^ test indicated that the distribution of several traits in both populations deviated significantly from normality. Corolla length (Figure 3) was notable in exhibiting high *χ* ^2^ values and correspondingly low *p‐*values in both populations, resulting from peaked and left‐skewed distributions. Overall, floral traits in both populations exhibited low to moderate *φ* coefficients (observed values, 0.10–0.55).

Rather than employing data transformations to normalize trait measurements, I used the nonparametric Spearman rank correlation coefficients (*r*_*s*_) to evaluate the strength of correlations between traits. Population differences in traits were evaluated with the nonparametric Mann–Whitney *U*‐test (Hollander & Wolfe, 1973). This test combines measurements from the White River and Norfork populations into a single sample and then assesses the range and location of the lowest group's distribution within the overall sample range against a ranked distribution that approaches normality. All *p*‐values are two‐tailed. To reduce the risk of Type 1 errors resulting from multiple comparisons, a Bonferroni correction was applied to similar analyses within each population. The significance level was adjusted by the number of tests, *α* = 0.05/6 = 0.0083.

## 3 RESULTS

### 3.1 Floral trait variation

Corolla length in the two populations ranged from 58 to 75 mm (Appendix 1). The minimum length represents the shortest distance that must be bridged by hummingbirds or bees to reach the nectary through the corolla opening. Median corolla length and opening width were 7.6% longer and 4.2% wider, respectively, in the White River population than at Norfork. Corolla length and width were marginally correlated at White River (*r*_*s*_ = 0.23, *p* = .01, *n* = 115) and less so at Norfork (*r*_*s*_ = 0.15, *p* = .09, *n* = 123). Differences in flower size between the two study sites may be partly attributed to site favorability, Norfork being higher and drier than White River.

The positions of the stigma, proximal anthers, and distal anthers, with respect to the nectary, were relatively similar in both populations (Appendix 1). Anthers and stigma were inserted well inside the corolla opening. The stigma was usually located between the proximal and distant pairs of anthers (Figure 3). The stricture‐nectary distance was similar in the two population samples (Mann–Whitney, *p*‐value = .83).

The proximal anther‐nectary distance exhibited a marginal correlation with corolla length at White River (*r*_*s*_ = 0.28, *p* = .002, *n* = 113), but a stronger correlation at Norfork (*r*_*s*_ = 0.48, *p* < .0001, *n* = 123). In both populations, there were strong correlations between corolla length and distal anther‐nectary distance (White River, *r*_*s*_ = 0.45, *p* < .0001, *n* = 113; Norfork, *r*_*s*_ = 0.53, *p* < .0001, *n* = 123). The stigma‐nectary distance was also significantly correlated with corolla length at White River (*r*_*s*_ = 0.37, *p* < .0001, *n* = 113) and Norfork (*r*_*s*_ = 0.26, *p* = .003, *n* = 123). All floral traits, external and internal, exhibited low coefficients of variation within populations (0.04–0.09).

### 3.2 Hummingbird foraging behavior

Ruby‐throats were observed at trumpet creeper flowers at the White River site from mid‐June to mid‐September. The frequency of visits increased in August coincident with the departure of orchard orioles (*Icterus spurius*), which compete for nectar and pierce flowers, and the augmentation of local hummingbird populations with hatching year birds. Ruby‐throats typically explore multiple inflorescences during foraging visits, making hovering passes around flower clusters before approaching closely. This surveillance likely serves multiple functions, including assessing bud development and flower maturity, locating oriole piercings as shortcuts to nectar, and checking for potential predators.

**FIGURE 4:**
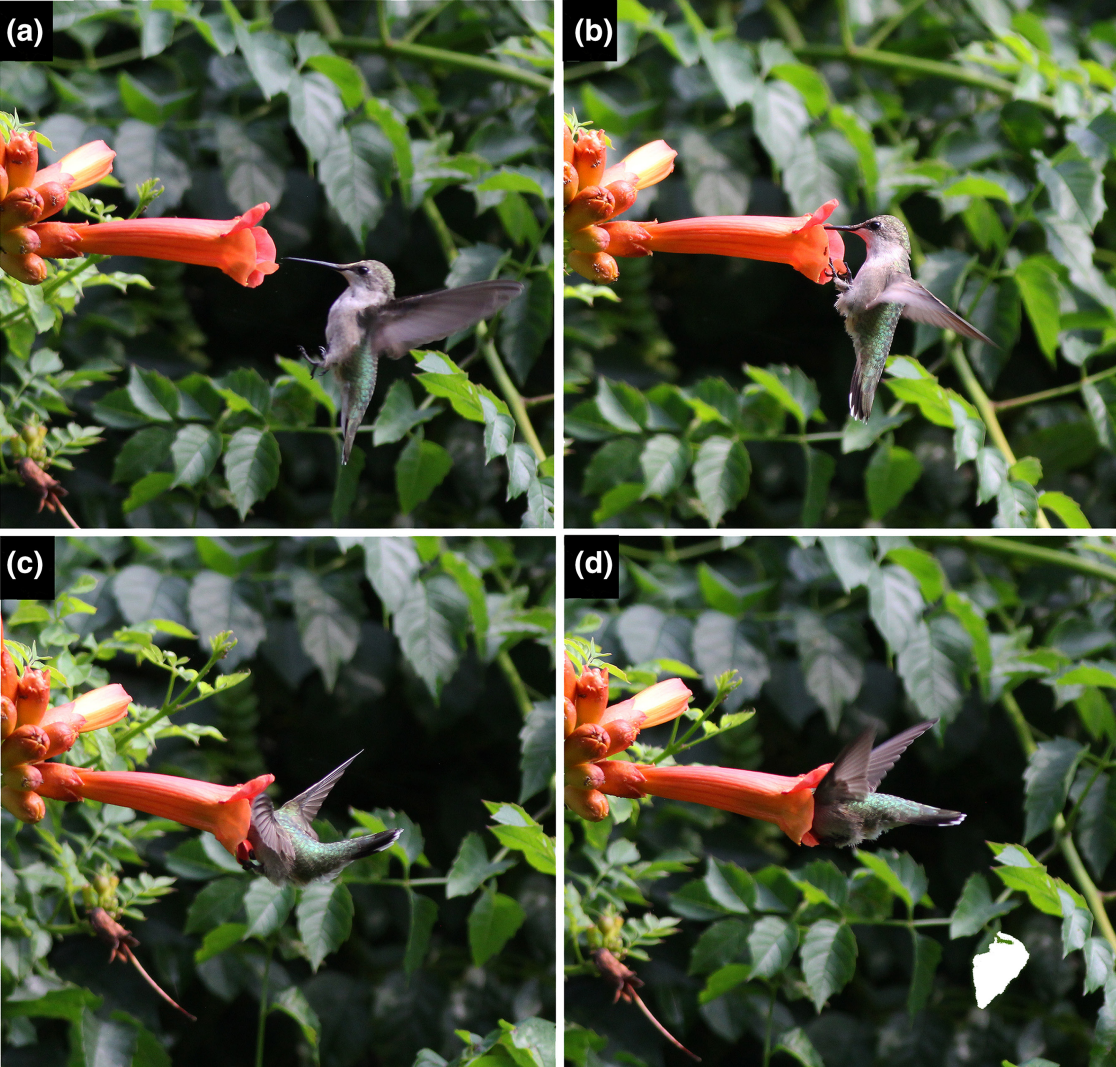
Ruby‐throated hummingbird floral‐diving at a newly‐opened trumpet creeper flower. (a) Inspection of flower and preparation to land. (b) Clinging to the ventral petal lobe with both feet. (c) Head and neck are thrust into the flower. (d) Wings paused while hummingbird extracts nectar.

After selecting a flower, a ruby‐throat hovers forward and grasps the ventral petal lobe at the corolla opening with both feet (Figure 4). The curved claws penetrate the soft spongy petal surface, providing leverage to thrust its head and upper body into the trumpet‐shaped corolla. When the fit is snug, individuals may become wedged or “caught” as observed by Catesby (1731). During these instances, wing movements typically pause, with the wings either folded or held in a posterior‐facing delta position (see video URL in Data Availability Statement). Ruby‐throats appear to employ leg thrusts and wing beats to back out of the corolla. Although scores of hummingbird species are known to insert the full length of their bills into flowers (see eBird photo archives, Media Search–Macaulay Library and eBird), until now, none have been documented to consistently thrust their entire head and upper body into snug tubular corollas while clinging. I propose the term “floral‐diving” to characterize this distinctive feeding technique, which was the only method ruby‐throats used to obtain nectar through the corolla opening at the Ozark study site.

As ruby‐throats enter and exit the corolla, their heads brush the stigma and both pairs of anthers, which are positioned well inside the flower opening. The snug fit and the position of the anthers near the dorsal midline ensure precise deposition of pollen on the hummingbird's crown. Individuals feeding at trumpet creeper flowers are dusted and often caked with pale yellow pollen (Bertin, 1982a). This foraging mode may require horizontal or upward‐facing flowers, as grasping the petal might be difficult from an inverted position (but see Reid et al., 2023). Hummingbirds may perform the floral‐diving maneuver 2–3 times in rapid succession at the same flower. These quick entrances and exits might reduce the risk of ambush by predators such as mantids (Nyffeler et al., 2017), vespine wasps (Carr & Golinski, 2020), and birds (Graves, 2022b), though this remains speculative.

Prolific flowering and the abundance of floral nectar and pollen make trumpet creeper highly appealing to hummingbirds and bees (Bertin, 1982a). However, the copious nectar is also irresistibly attractive to the nectar‐robbing orchard oriole (Graves, 2022a). Orioles use their bills to penetrate the corolla and the leathery calyx to access nectar. The slit‐like piercings typically measure 12–20 mm in length and extend through the base of the corolla parallel to the long axis of the flower. During the peak blooming season, orioles robbed more than 92% of flowers at the White River site before they departed in late July (Graves, 2022a). Hovering ruby‐throats readily visit oriole piercings and empty calyces after the corollas have been detached by orioles. While ruby‐throats are known secondary nectar‐robbers, there have been no documented observations of ruby‐throats piercing trumpet creeper corollas themselves, although this remains a possibility (see Inouye (1980) for discussion of primary and secondary nectar‐robbery).

### 3.3 Bill length and nectar accessibility

The conventional wisdom that corolla length of hummingbird‐pollinated plants matches the bill length of its primary pollinator (Dalsgaard et al., 2009; Maglianesi et al., 2014; Rico‐Guevara et al., 2021; Snow & Snow, 1980; Temeles et al., 2009) does not apply in this case. Trumpet creeper corollas are roughly three times the length of the longest bills of female (18.3 ± 0.7 mm) and male (16.4 ± 0.6 mm) ruby‐throats (bill measurements from Weidensaul et al., 2020; Temeles, 1996) used feeder experiments to demonstrate that the maximum depth of nectar extraction for female ruby‐throats, with fully extended tongues, was approximately 40 mm when feeding from 3 mm diameter tubes. This indicates that ruby‐throats must insert their bill tips about 36–53 mm past the corolla opening for their tongues to reach the circular nectary at the base of the tubular trumpet creeper flower. This distance may be a few millimeters less if nectar is pooled above the nectary, but the hummingbird's head must be fully inserted in either case. The eyes of ruby‐throats, located 22–24 mm behind the bill tip, are positioned 12–29 mm inside the tubular corolla during floral‐diving. While it is unclear whether their eyes are closed while feeding, the birds are certainly blinded and highly vulnerable to predators during this time.

## 4 DISCUSSION

One of the least anticipated findings was that ruby‐throats use their feet to cling to trumpet creeper flowers, enabling them to thrust their heads and upper bodies deeply into the snug corollas. While floral‐diving has not been formally reported in hummingbirds, it is likely that this foraging mode is practiced by several, perhaps many, hummingbird species in regions with rich ornithophilous floras. For example, a recent report on nectar‐robbery by the sword‐billed hummingbird (*Ensifera ensifera*) in the Ecuadorian Andes (Reid et al., 2023) included an unremarked photograph of a feeding bird clinging to the bottom of a hanging *Brugmansia sanguinea* flower. The bird's head is inserted into the corolla, while its wings are unencumbered and freely beating. Sword‐bills have long been known to insert their heads into hanging flowers of *Passiflora*, *Datura*, and *Brugmansia* while hovering (Fjeldså & Krabbe, 1990; Snow & Snow, 1980), but not while clinging. This example falls short of the immersive floral‐diving observed in ruby‐throats but it suggests that sword‐bills may be capable of similar behavior. These observations are emblematic of the major gaps in our knowledge of hummingbird foraging ecology. Such discoveries would be less surprising if they originated from remote sites in the species‐rich Andes (Rahbek & Graves, 2000), involving a poorly‐known, range‐restricted species (e.g., Fitzpatrick et al., 1979; Graves, 1980). However, if behaviors like clinging and floral‐diving can be overlooked in a well‐studied species in eastern North America, what else might we have missed?

The prevalence of clinging behavior in hummingbirds is not well understood. A recent survey by Colwell et al. (2023) categorized 220 species into two groups—those that cling to flowers with their feet to obtain nectar and those that obtain nectar solely by hovering. These groups were further subdivided into those that engage in nectar‐robbery and those that feed only legitimately. Clinging species tend to exhibit larger feet and shorter bills after accounting for body size, phylogenetic signal, and elevation. None of the 15 species that breed in the United States, including the ruby‐throat, has been reported to cling (Colwell et al., 2023). The ruby‐throat, which exhibits moderate foot size and bill length, was categorized as a hovering feeder that does not engage in nectar‐robbery.

Flower dissection revealed a second unexpected finding—the stricture in the trumpet creeper corolla is sufficiently narrow to prevent large bees from reaching the nectary. This constriction, situated 21–33 mm above the nectary, is buttressed by the swollen bases of the four staminal filaments and staminode. The aperture, blocked by the protruding style, forms a roughly concave diamond shape (insert in Figure 2). Ruby‐throats can easily insert their bill tip and tongue through the gap. Larger bees (e.g., *Apis*, *Bombus*, and *Xylocopa*) are unable to pass through the strictures of the largest trumpet creeper flowers. Several small halictid bees (*Augochlora* sp.) were found trapped in the narrow corolla base below the stricture during flower dissection. These were able to force their way through the stricture but were unable to back out.

The distance from the nectary to the stricture is 2–3 times longer than the proboscis length of the native bumblebees and carpenter bees in eastern North America (Cariveau et al., 2016), making the floral nectar inaccessible in uptilted flowers. Although the viscosity of 30% sucrose nectar at ambient temperatures (25–30°C) is relatively low (~2.1–2.2 mPa·s), it is unclear whether the nectar seeps into the slender basal stem and through the stricture of horizontally tilted flowers where long‐tongued bees could potentially reach it. In any event, the ruby‐throat appears to be the sole pollinator capable of easily accessing floral nectar in most trumpet creeper flowers.

These observations raise important questions about population variation in floral morphology and the prevalence of floral‐diving across the native range of trumpet creeper. The low coefficients of variation in trait measurements within the Ozark populations suggest that corolla length and the inserted positions of anthers and stigma may be under strong selection. However, there may be populations elsewhere with shorter flowers that ruby‐throats can feed from legitimately while hovering. Field observations have shown that nectar‐robbing orioles can disrupt pollination in local patches of trumpet creeper in the Ozarks (Graves, 2022a). The extent to which orioles undermine the mutualistic relationship between ruby‐throats and trumpet creeper in other areas where they geographically overlap remains to be determined. The function of nectar guides in trumpet creepers also calls for further study. Are they ancestral traits retained as a bet‐hedging strategy for bee‐pollination when hummingbird density is low, or do hummingbirds use them to pinpoint the corollary stricture? Do they vary geographically? Nectar guides in narrow tubular corollas are likely hidden from view of nectar‐feeding birds, but they may serve a purpose in spacious campanulate flowers where converging lines are easily seen by ruby‐throats entering the corolla. Other pressing tasks include a geographic assessment of floral nectar volumes, the concentrations of disaccharide and monosaccharide sugars, and the potential use of extrafloral nectaries by hummingbirds. Finally, kinematic analysis of clinging and floral‐diving may provide valuable insight into the energetic costs and benefits of this immersive and apparently rare foraging mode in hummingbirds.

## AUTHOR CONTRIBUTIONS

**Gary R. Graves:** Conceptualization (lead); data curation (lead); formal analysis (lead); funding acquisition (lead); investigation (lead); methodology (lead); project administration (lead); resources (lead); supervision (lead); validation (lead); visualization (lead); writing – original draft (lead); writing – review and editing (lead).

## FUNDING INFORMATION

None.

## CONFLICT OF INTEREST STATEMENT

The author has no conflict of interest to declare.

## Supporting information

Appendix S1

## Data Availability

Raw data for floral trait metrics are deposited in Dryad https://doi.org/10.5061/dryad.k6djh9wgp. Video of hummingbird foraging is available at ML617831323‐Ruby‐throated Hummingbird‐Macaulay Library.

## APPENDIX 1 SUMMARY STATISTICS OF FLORAL TRAIT MEASUREMENTS (MM) OF *CAMPSIS RADICANS* FROM TWO POPULATIONS IN THE OZARK MOUNTAINS IN NORTHERN ARKANSAS

**Table:**

			White River	Norfork	Mann–Whitney *U*	
					*Z*	*p*
	***n***		**115**	**123**		
Corolla length	Mean (SD)		70.1 ± 3.1	65.4 ± 2.5		
	CV		0.04	0.04		
	Median		70.6	65.6	10	<.001
	Range		60.2–75.2	57.6–70.8		
	Kurtosis		0.53	1.22		
	Skewness		−0.94	−0.80		
	D'Agostino's K2 test	*χ* ^2^	15.7	16.20		
		*p*	<.001	<.001		
		*φ*	0.37	0.36		
	***n***		**114**	**123**		
Stricture‐nectary distance	Mean (SD)		25.0 ± 1.8	24.9 ± 1.8		
	CV		0.07	0.07		
	Median		25	25	0.21	.83
	Range		21–33	21–30		
	Kurtosis		2.39	−0.16		
	Skewness		0.65	0.44		
	D'Agostino's K2 test	*χ* ^*2*^	17.3	4.1		
		*p*	<.001	.13		
		*φ*	0.39	0.18		
	***n***		**114**	**123**		
Proximal anthers‐nectary distance	Mean (SD)		53.6 ± 2.5	52.9 ± 2.9		
	CV		0.05	0.06		
	Median		54	53	2.34	.02
	Range		47–63	43–61		
	Kurtosis		1.24	0.47		
	Skewness		0.14	0.1		
	D'Agostino's K2 test	*χ* ^*2*^	5.0	1.5		
		*p*	.08	.47		
		*φ*	0.21	0.11		
	***n***		**114**	**123**		
Distal anthers‐nectary distance	Mean (SD)		60.3 ± 2.6	60.5 ± 2.6		
	CV		0.04	0.04		
	Median		60	61	0.42	.68
	Range		50–67	53–65		
	Kurtosis		1.81	0.16		
	Skewness		−0.59	−0.46		
	D'Agostino's K2 test	*χ* ^*2*^	13.5	4.6		
		*p*	.001	.10		
		*φ*	0.34	0.19		
	***n***		**114**	**123**		
Stigma‐nectary distance	Mean (SD)		54.4 ± 3.2	53.8 ± 4.0		
	CV		0.06	0.07		
	Median		54	53	2.51	.01
	Range		43–64	45–70		
	Kurtosis		2.11	3.23		
	Skewness		−0.62	1.21		
	D'Agostino's K2 test	*χ* ^*2*^	15.7	35.9		
		*p*	>.001	<.0001		
		*φ*	0.37	0.54		
	***n***		**114**	**123**		
Corolla opening width	Mean (SD)		19.6 ± 1.5	18.8 ± 1.7		
	CD		0.08	0.09		
	Median		19.7	18.9	3.66	<.001
	Range		16.1–22.8	13.5–24.0		
	Kurtosis		−0.52	1.72		
	Skewness		−0.10	−0.61		
	D'Agostino's K2 test	*χ* ^*2*^	2.20	14.4		
		*p*	.33	<.001		
		*φ*	0.14	0.34		
