# Supplementary material for: Ornithophily in the trumpet creeper (Campsis radicans)
Source: Ecol Evol. 2024 Sep 10;14(9):e70279. doi: 10.1002/ece3.70279 (PMC11387201; doi:10.1002/ece3.70279)
Supplement: Supplementary file 1 — Appendix S1 [file ECE3-14-e70279-s001.pdf]

Field number	Date (2023)	Location	Corolla length	Corolla width at mouth	Nectary-stricture distance	Nectary-proximal anther distance	Nectary-distal anther distance	Nectary-stigma distance
37	4-Aug	Norfork	66.3	18.3	24	52	61	56
38	4-Aug	Norfork	58	19.1	21	43	53	48
39	4-Aug	Norfork	66.7	17.4	24	55	57	52
40	4-Aug	Norfork	62	18.8	24	51	60	52
41	4-Aug	Norfork	65.4	18.3	23	51	57	52
42	4-Aug	Norfork	65.7	18.3	23	51	61	54
43	4-Aug	Norfork	66.7	20.8	23	54	62	54
44	4-Aug	Norfork	64.8	19.8	22	49	58	52
45	4-Aug	Norfork	68.5	21.7	25	55	64	59
46	4-Aug	Norfork	66.1	19.3	24	52	62	56
47	4-Aug	Norfork	66.8	18.1	25	56	63	53
48	4-Aug	Norfork	67.9	19.2	25	53	62	57
49	4-Aug	Norfork	67.3	21.4	24	49	58	53
50	4-Aug	Norfork	66.6	21.3	25	55	59	54
51	4-Aug	Norfork	67.8	20.3	23	50	57	51
52	4-Aug	Norfork	65.2	18.5	24	50	59	52
53	4-Aug	Norfork	67.5	18.2	27	55	63	56
54	4-Aug	Norfork	66.4	19.2	25	53	60	55
55	4-Aug	Norfork	68.1	19.5	27	51	60	56
56	4-Aug	Norfork	65.3	18.4	25	52	59	54
57	4-Aug	Norfork	67.1	18.3	28	54	62	55
58	4-Aug	Norfork	64.3	19.4	24	53	61	50
59	4-Aug	Norfork	59.1	18.5	22	49	56	53
60	4-Aug	Norfork	65	19.8	25	52	61	54
61	4-Aug	Norfork	67.7	18.4	24	59	63	53
62	4-Aug	Norfork	66.1	19.1	25	55	61	49
63	4-Aug	Norfork	67.6	20.2	23	51	60	50
64	4-Aug	Norfork	64.6	19.7	23	55	60	54
65	4-Aug	Norfork	66.5	19.5	25	54	62	57
66	4-Aug	Norfork	64.6	17.2	25	53	58	47
67	4-Aug	Norfork	64.6	19.2	24	50	61	51
68	4-Aug	Norfork	65.4	21.2	23	50	58	52
69	4-Aug	Norfork	66.4	19.7	26	54	62	55
72	6-Aug	Norfork	62.8	17.3	26	52	59	53
73	7-Aug	Norfork	63	18.5	25	51	59	53
74	8-Aug	Norfork	66.6	18.4	29	59	65	56
75	6-Aug	Norfork	65.4	19.3	24	51	60	56
76	7-Aug	Norfork	64.5	18.6	28	55	61	51
77	8-Aug	Norfork	65.8	18.5	26	55	60	55
78	6-Aug	Norfork	63.8	18.9	24	51	58	55
79	7-Aug	Norfork	65.3	18.4	26	54	61	55
80	8-Aug	Norfork	67.6	20	27	53	65	59

81	6-Aug	Norfork	64.4	19.9	25	51	58	52
82	7-Aug	Norfork	67.7	17.3	28	56	65	56
83	8-Aug	Norfork	65.2	19	28	58	64	57
84	6-Aug	Norfork	65.1	17	26	54	61	52
85	7-Aug	Norfork	65.6	19	24	51	59	53
86	8-Aug	Norfork	61.2	19.2	24	50	58	53
87	6-Aug	Norfork	65.2	18	27	55	62	52
88	7-Aug	Norfork	65.3	18	26	56	64	52
89	8-Aug	Norfork	65.3	20.2	25	52	60	54
90	6-Aug	Norfork	63.9	16.7	28	54	62	53
91	6-Aug	Norfork	66	18.4	25	51	58	53
92	6-Aug	Norfork	65.7	20	24	51	59	55
93	6-Aug	Norfork	63.8	17.6	24	51	59	56
94	6-Aug	Norfork	64.5	17.4	27	56	62	58
95	6-Aug	Norfork	64.6	18.5	24	52	59	55
96	6-Aug	Norfork	61.8	19.7	23	49	56	51
97	6-Aug	Norfork	63.1	19.5	23	52	59	54
98	8-Aug	Norfork	66.8	16.3	29	55	63	52
99	8-Aug	Norfork	64.7	18.6	25	49	57	57
100	8-Aug	Norfork	65	18.2	25	57	62	53
101	8-Aug	Norfork	57.6	17.9	23	49	57	45
102	8-Aug	Norfork	66.3	19.6	26	55	60	49
103	8-Aug	Norfork	64.5	18.1	23	51	58	52
151	14-Aug	Norfork	67	18.9	30	55	63	53
152	14-Aug	Norfork	65.8	18.3	27	58	63	54
153	14-Aug	Norfork	64.3	20.2	24	59	63	52
154	14-Aug	Norfork	64.4	16.1	26	52	62	53
155	14-Aug	Norfork	65.8	16.3	28	58	63	56
161	14-Aug	Norfork	68.4	19.6	25	54	63	52
162	14-Aug	Norfork	66.3	19.1	24	55	64	56
163	14-Aug	Norfork	63.6	17.7	28	59	66	55
164	14-Aug	Norfork	68.8	19.9	26	58	63	55
165	14-Aug	Norfork	69.5	19.1	27	55	65	59
206	22-Aug	Norfork	70.8	14.7	24	57	65	65
207	22-Aug	Norfork	66.8	15	26	54	62	66
208	22-Aug	Norfork	66.1	13.8	24	51	60	63
209	22-Aug	Norfork	64.9	18.4	23	50	61	55
210	22-Aug	Norfork	67.6	18.4	28	57	63	49
211	22-Aug	Norfork	70	20.3	26	53	64	58
212	22-Aug	Norfork	69.3	13.5	26	56	64	59
213	22-Aug	Norfork	66.1	13.5	25	53	60	63
214	22-Aug	Norfork	69.9	18.5	26	61	63	50
215	22-Aug	Norfork	64.8	18.8	24	52	59	53
216	22-Aug	Norfork	64.5	18.9	23	50	57	52
217	22-Aug	Norfork	67.1	18	26	53	60	54
246	30-Aug	Norfork	65.4	18.9	25	49	59	55
247	30-Aug	Norfork	68.1	21.5	23	50	61	51

248	30-Aug	Norfork	68	20.4	25	52	62	55
249	30-Aug	Norfork	66.6	16.6	29	54	62	49
250	30-Aug	Norfork	68.7	19.5	28	56	62	54
251	30-Aug	Norfork	67.4	17.4	26	56	62	50
252	30-Aug	Norfork	64.4	15.3	26	51	60	48
253	30-Aug	Norfork	59.1	17.1	22	48	54	50
254	30-Aug	Norfork	61.1	18.8	26	50	60	57
255	30-Aug	Norfork	63	18.8	22	50	57	61
258	30-Aug	Norfork	62.1	15.9	26	53	62	70
259	30-Aug	Norfork	63	19.1	24	51	58	48
260	30-Aug	Norfork	67.7	19.1	24	56	63	55
261	30-Aug	Norfork	63.7	18	25	52	62	68
262	30-Aug	Norfork	66.2	19.7	23	55	61	50
263	30-Aug	Norfork	66	18.9	26	54	61	53
264	30-Aug	Norfork	66.1	21.2	22	50	57	50
265	30-Aug	Norfork	70.4	20.6	27	53	60	52
266	30-Aug	Norfork	68.5	21.5	25	53	62	54
267	30-Aug	Norfork	63.8	20.8	24	49	58	53
268	30-Aug	Norfork	65.9	16.9	26	53	60	49
269	30-Aug	Norfork	68.4	24	26	53	62	53
270	30-Aug	Norfork	64.1	18.8	23	52	57	51
276	30-Aug	Norfork	66.8	17.2	25	54	63	57
277	30-Aug	Norfork	66.5	21.5	23	54	61	54
278	30-Aug	Norfork	66	20.9	23	50	58	55
279	30-Aug	Norfork	64.4	20.4	23	49	58	52
280	30-Aug	Norfork	67.4	20.7	24	52	59	54
315	6-Sep	Norfork	65.6	21.2	25	53	62	53
316	6-Sep	Norfork	62.9	19.8	24	50	60	52
317	6-Sep	Norfork	64.5	19.1	22	55	62	45
318	6-Sep	Norfork	61.7	19.7	26	53	62	48
320	20-Sep	Norfork	62.4	17.5	26	54	62	50
321	20-Sep	Norfork	58.2	19.4	23	47	54	49
322	20-Sep	Norfork	60.7	20.5	24	51	57	50
323	20-Sep	Norfork	59	19.8	23	48	53	59
1	29-Jul	White River	70.4	21.4	22	58	67	64
2	29-Jul	White River	67.7	19.7	27	54	61	58
3	29-Jul	White River	70.1	19.5	28	55	61	53
4	29-Jul	White River	73.7	18.9	28	57	64	60
5	29-Jul	White River	72.6		27	55	61	58
6	30-Jul	White River	69.7	20.2	26	50	58	51
7	30-Jul	White River	72.6	18.3	25	52	60	52
8	30-Jul	White River	69.1	19.1	26	53	59	54
9	30-Jul	White River	72	18.7	26	55	64	59
10	30-Jul	White River	69.7	17.9	25	49	53	51
11	31-Jul	White River	67.4	20.6	25	58	63	59
12	31-Jul	White River	72.4	22	24	55	59	56
13	31-Jul	White River	72.2	20.1	33	56	61	54

14	31-Jul	White River	68.3	19.1	25	54	60	56
15	31-Jul	White River	60.2	18.5	24	49	55	51
16	31-Jul	White River	70.9	18.5	26	54	63	59
17	1-Aug	White River	65.9	18	25	51	55	49
18	1-Aug	White River	70.3	20	24	57	64	60
19	1-Aug	White River	63.1	18.2	23	50	56	54
20	1-Aug	White River	69.5	20.3	25	52	61	56
21	1-Aug	White River	71.3	20.7	27	53	60	56
22	1-Aug	White River	62.9	17.3	23	52	56	52
23	1-Aug	White River	66.4	17.5	23	53	60	54
24	1-Aug	White River	70	19.8	23	52	60	57
25	2-Aug	White River	66.8	20.6	26	54	59	52
26	2-Aug	White River	69.6	19.4	26	55	62	59
27	2-Aug	White River	66.6	18.5	24	58	61	55
28	2-Aug	White River	69.6	18.4	24	52	59	56
29	2-Aug	White River	71.6	20.7	24	52	60	56
30	3-Aug	White River	72.3	17.4	24	52	60	54
31	3-Aug	White River	70.2	20.7	23	54	60	56
32	3-Aug	White River	70.4	19.3	24	51	57	53
33	3-Aug	White River	65.9	19.6	22	53	58	54
34	3-Aug	White River	71.1	17.7	25	56	63	56
35	3-Aug	White River	64.4	18.5	26	55	61	57
36	3-Aug	White River	70.1	17.6	25	51	60	53
70	5-Aug	White River	67.9	19.6	24	47	55	47
71	5-Aug	White River	62.9	22.6	25	47	50	44
104	9-Aug	White River	69.4	20.1	26	50	58	53
105	9-Aug	White River	72.6	20.2	27	53	60	54
106	9-Aug	White River	72.1	22	26	50	59	52
107	9-Aug	White River	72.7	20.3	27	55	62	54
108	9-Aug	White River	61.9	20.9	22	52	58	54
109	9-Aug	White River	73	20.1	27	56	63	59
146	10-Aug	White River	72.8	20.8	26	53	58	54
147	10-Aug	White River	73.4	21.5	25	57	61	54
148	10-Aug	White River	72.5	22.5	25	55	63	56
149	10-Aug	White River	72.2	22	24	50	60	56
150	10-Aug	White River	72.8	19.5	23	55	60	49
156	15-Aug	White River	73.9	21.4	26	53	60	52
157	15-Aug	White River	70.3	18.6				
158	15-Aug	White River	70.6	19.5	27	55	65	60
159	15-Aug	White River	69.5	20.5	25	54	61	55
160	15-Aug	White River	72.1	21.8	27	57	60	55
166	16-Aug	White River	69.9	20.1	26	50	59	55
167	16-Aug	White River	71.5	20.1	23	53	58	54
168	16-Aug	White River	71.6	21.1				
169	16-Aug	White River	68	16.7	26	51	59	49
170	16-Aug	White River	73.2	17.4	26	56	62	54
171	17-Aug	White River	69.2	19.4	24	52	60	54

172	17-Aug	White River	73.2	20.6	28	52	63	59
173	17-Aug	White River	70	20.4	25	50	57	52
174	17-Aug	White River	71.4	21.8	25	54	61	56
175	17-Aug	White River	68.8	20.9	22	55	59	53
201	22-Aug	White River	69.2	18.7	23	54	58	50
202	22-Aug	White River	73.5	20.7	26	53	63	57
203	22-Aug	White River	73.4	20.2	25	51	61	55
204	22-Aug	White River	68	18.1	23	54	62	53
205	22-Aug	White River	72.9	19.9	23	51	58	52
223	24-Aug	White River	71.2	18.2	22	50	56	50
224	24-Aug	White River	68.6	16.1	25	55	59	54
225	24-Aug	White River	71	20.1	24	52	60	54
226	25-Aug	White River	70.8	17.3	25	54	60	54
227	25-Aug	White River	70.2	19.3	26	56	58	55
228	25-Aug	White River	69.4	20.2	25	51	59	52
229	25-Aug	White River	72.1	18.6	27	56	62	57
230	25-Aug	White River	66	18	25	52	59	45
257	30-Aug	White River	65.8	18	22	56	60	51
271	1-Sep	White River	66.7	16.9	24	55	61	52
272	1-Sep	White River	65	19.3	23	53	60	53
273	1-Sep	White River	65.5	16.3	24	51	55	50
274	1-Sep	White River	64.7	19.5	22	54	59	54
275	1-Sep	White River	68.8	18.8	21	51	59	52
281	2-Sep	White River	62.6	18.8	23	53	61	52
282	2-Sep	White River	71.8	21.7	25	55	63	55
283	2-Sep	White River	70	20.7	26	56	63	55
284	3-Sep	White River	70.9	21.6	25	54	60	53
285	3-Sep	White River	65.7	19.8	27	56	63	55
286	3-Sep	White River	70.9	20.6	27	54	62	54
287	3-Sep	White River	71.1	20.3	23	53	58	52
288	3-Sep	White River	74.1	21	27	53	60	53
289	3-Sep	White River	73.3	17.1	27	63	67	58
290	3-Sep	White River	70.2	18.7	22	55	63	55
291	3-Sep	White River	71.8	20.4	26	56	63	55
292	3-Sep	White River	73.1	18.4	24	54	61	54
293	4-Sep	White River	75.2	20.5	28	56	64	58
294	4-Sep	White River	71.1	19.2	24	54	62	55
295	4-Sep	White River	71.8	17.9	25	59	63	57
296	4-Sep	White River	73.7	18.5	23	54	64	59
297	4-Sep	White River	73.4	19.3	25	55	63	58
298	4-Sep	White River	72.6	18.4	23	55	63	57
299	5-Sep	White River	74.3	20.5	25	54	60	54
300	5-Sep	White River	73.6	22.5	24	56	61	56
301	5-Sep	White River	74.9	21.5	27	55	62	54
302	5-Sep	White River	74.8	20	26	54	62	56
303	5-Sep	White River	72.6	18.9	26	54	64	58
304	6-Sep	White River	73.8	22.8	24	57	64	57

305	6-Sep	White River		21.1	28	54	60	56
306	6-Sep	White River	72.5	18.4	26	53	59	54
307	6-Sep	White River	70.8	17.9	25	53	61	56
308	9-Sep	White River	69.9	21.2	26	50	59	43
309	9-Sep	White River	68.4	20.4	26	52	58	53
310	9-Sep	White River	70.5	20.8	24	55	61	55
314	10-Sep	White River	71.2	20.4	23	54	61	56
319	20-Sep	White River	67.6	19	25	52	59	54
256	30-Aug	White River	63.4	16.9	23	56	62	56
